# Functional development of carbon dioxide detection in the maxillary palp of *Anopheles gambiae*

**DOI:** 10.1242/jeb.116798

**Published:** 2015-08

**Authors:** Bonaventure Aman Omondi, Shahid Majeed, Rickard Ignell

**Affiliations:** 1 Unit of Chemical Ecology, Department of Plant Protection Biology, Swedish University of Agricultural Sciences, Alnarp 230 53, Sweden; 2 Department of Evolutionary Neuroethology, Max Planck Institute for Chemical Ecology, Hans-Knoell-Strasse 8, Jena 07745, Germany

**Keywords:** Gene expression, Modulation, Host seeking, Behaviour, Physiology, Mosquito

## Abstract

Olfactory information drives several behaviours critical for the survival and persistence of insect pests and vectors. Insect behaviour is variable, linked to their biological needs, and regulated by physiological dynamics. For mosquitoes, CO_2_ is an important cue that signifies the presence of a host, and which elicits activation and attraction. To investigate the genetic basis of olfactory modulation in mosquitoes, we assayed changes in CO_2_ detection from receptor gene expression through physiological function to behaviour, associated with the onset of host seeking in the malaria vector, *Anopheles gambiae*. The gene encoding a subunit of the CO_2_ receptor, *AgGr22*, was found to be significantly up-regulated in host-seeking females, consistent with a significant increase in sensitivity of CO_2_-responsive neurons (cpA) housed in capitate peg sensilla of the maxillary palp. In addition, the odorant receptor *AgOr28*, which is expressed in cpC neurons, was significantly up-regulated. In contrast, *AgOr8*, which is expressed in cpB neurons, was not affected by this change in physiological state, in agreement with results for the obligate co-receptor *Orco*. Moreover, the sensitivity of the cpB neuron to (*R*)-1-octen-3-ol, a well-known mammalian kairomone, did not change in response to the onset of host seeking. The concentration of CO_2_ flux influenced both the propensity of *A. gambiae* to take off into the wind and the speed with which this activation occurred. Female *A. gambiae* mosquitoes responded to CO_2_ whether mature for host seeking or not, but onset of host seeking enhanced sensitivity and speed of activation at relevant doses of CO_2_.

## Abstract (precis)

**Summary:** Onset of host-seeking behaviour in malaria mosquitoes is correlated with an increased receptor gene expression, and physiological and behavioural sensitivity to CO_2_.

## INTRODUCTION

Olfaction plays a vital role in the location and discrimination of resources in insects, and is a candidate target for sustainable pest control (Carey and Carlson, 2011; Pask et al., 2013; Tauxe et al., 2013). Blood-feeding insects, such as the African malaria mosquito, *Anopheles gambiae*, respond to plant volatiles and emanations from their potential blood hosts, including metabolic by-products of animals and their cutaneous microbes (Bohbot et al., 2010; Foster and Takken, 2004; Takken and Knols, 1999; Verhulst et al., 2010). The behavioural response to these cues is not static but dependent on endogenous regulatory mechanisms related to the physiological state of the insect (Bohbot et al., 2013; Brown et al., 1994; Grant and O'Connel, 2007; Nyasembe et al., 2014). For example, upon eclosion, female *A. gambiae* do not seek blood hosts for up to 24–48 h, after which they will readily orient towards such hosts and take a blood meal (Foster and Takken, 2004). Mating is not a prerequisite for blood feeding but influences egg development in blood-fed females (Lounibos, 1994; Verhulst et al., 2010). Following a successful blood meal, these mosquitoes again ignore potential sources of a blood meal until after egg laying (Anton et al., 2007; Klowden and Briegel, 1994; Klowden and Lea, 1979a,b, 1998; Qiu et al., 2013; Takken et al., 2001). Such physiological changes provide a practical model for studying olfactory modulation in insects, especially in mosquitoes (Anton et al., 2007; Rinker et al., 2013a,b; Saveer et al., 2012). Moreover, as blood-feeding preference is a key determinant of the epidemiological role of mosquitoes as disease vectors, an understanding of its modulation has important implications for human and animal health (Carey and Carlson, 2011; Cohuet et al., 2011; Potter, 2014).

Carbon dioxide (CO_2_), emitted by all potential blood hosts, is a key kairomone for mosquitoes, which signifies the presence of a blood source and sensitises them to other host sensory cues (Dekker et al., 2005; Gillies, 1980; McMeniman et al., 2014; Webster et al., 2015). Activation to CO_2_ is a component of source searching, which would make the mosquito more liable to detect the source given other odours (Dekker et al., 2005; Webster et al., 2015). CO_2_ is an attractant in itself, but also synergises with host odours and primes take-off, sustained ﬂight behaviour and landing in host-seeking mosquitoes (Costantini et al., 1996; Spitzen et al., 2008; Webster et al., 2015). Flowers also emit CO_2_; however, the role of this compound in floral quality evaluation in teneral stages of mosquitoes has not received particular attention, when compared with other non-blood-feeding species of insect, e.g. moths (Thom et al., 2004). Detection of CO_2_ with the heteromeric gustatory receptor system is basal in several insect orders, and well conserved among insects (Robertson and Kent, 2009). In *A. gambiae*, three subunits (*AgGr22*, *AgGr23* and *AgGr24*) function together to mediate CO_2_ detection (Lu et al., 2007). Functional analyses of these genes through heterologous expression (Lu et al., 2007), gene knock-out (McMeniman et al., 2014) and transient knockdown of orthologous *Gr*s in the yellow fever mosquito, *Aedes aegypti* (Erdelyan et al., 2012), have suggested a conserved role of these genes as CO_2_ receptors. They are expressed in one of the olfactory sensory neurons (OSNs), referred to as cpA, within the capitate peg sensilla on the maxillary palp of mosquitoes (Grant et al., 1995; Lu et al., 2007). In *A. aegypti*, these neurons exhibit an age-dependent increase in sensitivity, suggesting that changes in the sensory capability of the system are timed to occur with the onset of host-seeking behaviour (Grant and O'Connell, 2007). Similar changes in OSN sensitivity have been observed in the cpB neuron of *A. aegypti*, which expresses the odorant receptor *Or8* along with the canonical receptor *Orco*. The latter receptor is tuned to (*R*)-1-octen-3-ol, a kairomone cue emitted by most mammals (Bohbot et al., 2013).

Transcription profiling has been used to infer the function and modulation of several insect receptors, where mRNA transcript abundance has been linked with protein (receptor) function (Abrieux et al., 2013; Bohbot et al., 2013; Iatrou and Biessmann, 2008; Poivet et al., 2013; Rinker et al., 2013a,b). Although the sensitivity of the OSNs detecting CO_2_ and (*R*)-1-octen-3-ol has been shown to increase with age in *A. aegypti* (Bohbot et al., 2013; Grant and O'Connell, 2007), the relationship between transcript abundance and behaviour has yet to be investigated. We hypothesised that higher transcription would lead to increased sensitivity to a ligand, a stronger response or a wider dynamic range. This would enhance the insect's ability to detect and track fluctuations in the concentration of the ligand it perceives. In this study, we assayed gene expression and odorant detection in the maxillary palp system of *A. gambiae* to evaluate the molecular, physiological and behavioural modulation of odorant reception.

## RESULTS

### Gene regulation

**Fig. 1.**
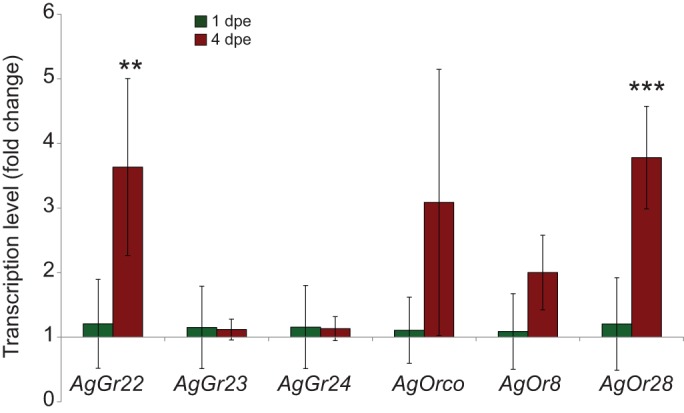
**Expression of the CO_2_ receptor repertoire significantly increases with age.** The relative transcription levels (means±s.d.) of CO_2_ receptor genes (*AgGr22*, *AgGr23* and *AgGr24*) and odorant receptor genes (*AgOrco*, *AgOr8* and *AgOr28*) in 1 and 4 day old (1 and 4 days post-eclosion, dpe) *Anopheles gambiae*. Relative transcription level increases significantly (**P<0.01) for *AgGr22* but not for the other two subunits, and for *AgOr28* but not for the other *AgOr* transcripts over the same period.

Real-time PCR assay showed that of the three CO_2_ receptor subunits, only *AgGr22* transcripts were significantly enhanced in 4 day old (4 days post-eclosion, dpe) relative to 1 dpe mosquitoes (Fig. 1; *t*=5.254, *P*≤0.003, d.f.=5). Similarly, odorant receptor *AgOr28* transcripts were significantly enhanced in 4 dpe compared with 1 dpe mosquitoes (Fig. 1; *t*=6.746, *P*≤0.001, d.f.=5); changes in the other odorant receptor transcripts were not statistically significant between age groups (Fig. 1; *AgOr8*: *t*=2.304, *P*≤0.069, d.f.=5; and *Orco*: *t*=2.294, *P*≤0.070, d.f.=5).

### Neural activity

**Fig. 2.**
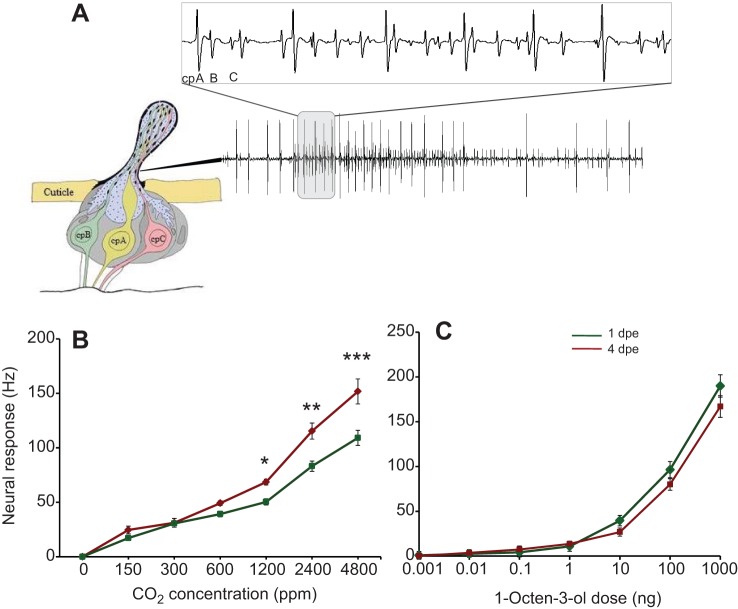
**The sensitivity of the CO_2_ olfactory sensory neurons (OSNs) is correlated with receptor transcription.** (A) The capitate peg sensillum houses three neurons, classified according to spike amplitude: cpA expresses the three subunits, *Gr22*–*24*, that mediate CO_2_ detection, cpB expresses *Or8* and *Orco*, which respond to (*R*)-1-octen-3-ol, while cpC expresses *Or28* and *Orco*. (B) The sensitivity of cpA to CO_2_ is increased in 4 dpe relative to 1 dpe mosquitoes. (C) cpB does not change its sensitivity to (*R*)-1-octen-3-ol over the same time period. *P<0.05, **P<0.01, ***P<0.001.

Single sensillum recordings (Fig. 2A) showed a significantly enhanced response to CO_2_ in 4 dpe relative to 1 dpe mosquitoes at all concentrations above 600 ppm, with a significant interaction between age and treatment (*F*_5,108_=4.83, *P*≤0.0005; Fig. 2B). Moreover, 4 dpe mosquitoes had a lower CO_2_ detection threshold than 1 dpe mosquitoes (Fig. 2B). Detection threshold and strength of response to (*R*)-1-octen-3-ol were not significantly different between 1 and 4 dpe mosquitoes (Fig. 2C).

### Activation by CO_2_

**Fig. 3.**
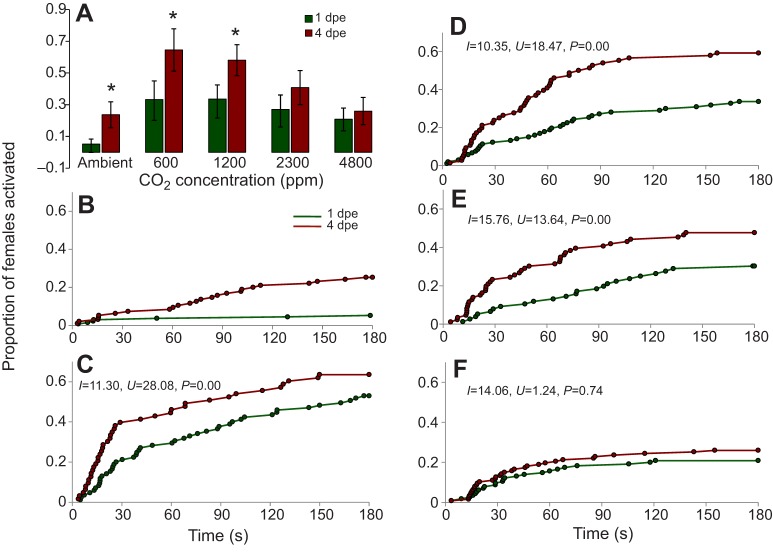
**Activation profiles of 1 and 4** **dpe *A. gambiae* females by CO_2_ flux is dependent on the concentration of CO_2_.** (A) Both 1 and 4 dpe mosquitoes are activated by CO_2_ but at different rates, both with an optimum range of 600–1200 ppm. Error bars represent the 95% confidence interval for binomial probability for each result (see also Table 2). *P<0.05. (B–F) Stimulation by CO_2_ above ambient levels increased the propensity and speed of activation [B, 380 ppm (ambient); C, 600 ppm; D, 1200 ppm; E, 2400 ppm; F, 4800 ppm]. Most mosquitoes take off as soon as CO_2_ is detected, giving a positive skew to the response–time function relative to the diagonal line (describing uniform interval activation).

In the bioassay, optimum CO_2_ activation occurred between 600 and 1200 ppm for both age classes, but 4 dpe mosquitoes were more responsive to CO_2_ stimulation at these concentrations (Fig. 3A). At ambient CO_2_ stimulation, the activation pattern was similar to random uniform flight with only <5% of mosquitoes activated (Cox–Mantel test, *I*=5.55, *U*=0.71, *P*≤0.76; and *I*=3.21, *U*=−0.70, *P*≤0.69 for 1 and 4 dpe mosquitoes, respectively; Fig. 3B). Enhanced levels of CO_2_ above ambient concentration, however, resulted in both a greater proportion of mosquitoes activated and faster instantaneous activation of both age classes compared with random uniform activation, resulting in activation functions described by a convex-shaped line compared with the hypothetical diagonal line between the origin and maximum activation (Fig. 3C,D, Table 1). A significantly higher proportion of 4 dpe mosquitoes were activated by all CO_2_ treatment levels, but at 4800 ppm CO_2_ a lower activation rate occurred, resulting in a diminished difference between the two age classes (Fig. 3).

**Table 1.**
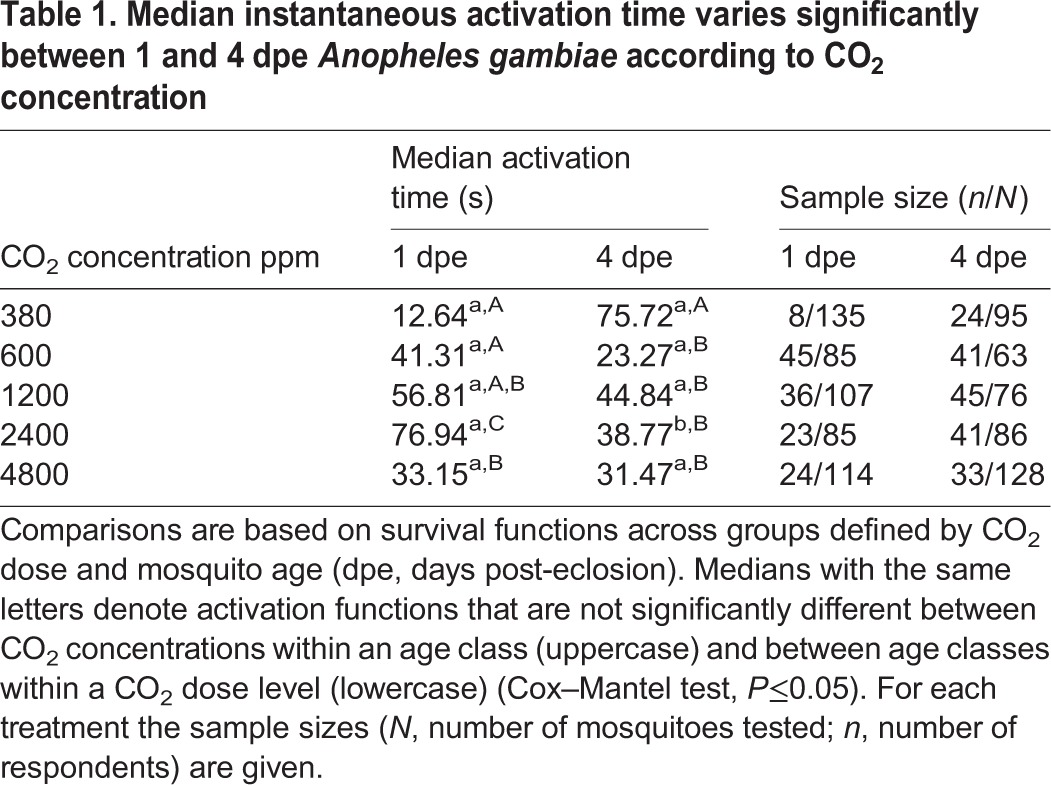
**Median instantaneous activation time varies significantly between 1 and 4 dpe *Anophelesgambiae* according to CO_2_ concentration**

**Table 2.**
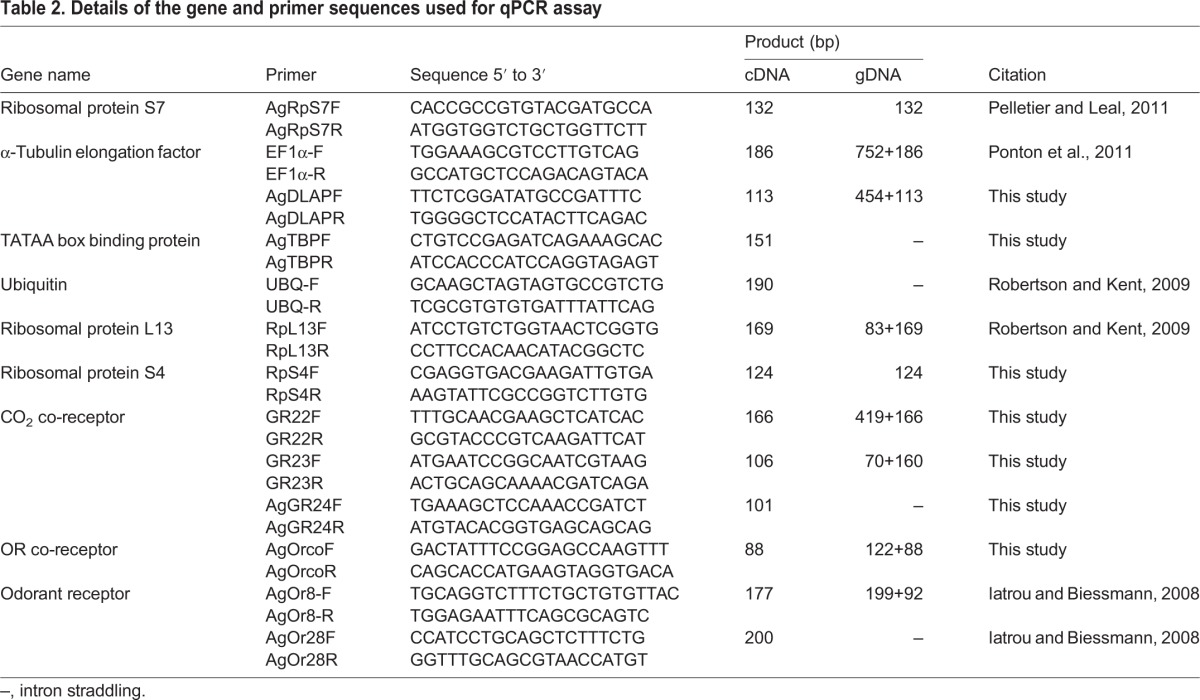
**Details of the gene and primer sequences used for qPCR assay**

## DISCUSSION

The olfactory receptors expressed in the maxillary palps of *A. gambiae* are active within 24 h of emergence, but undergo transcriptional changes as the mosquito matures for blood-host seeking. Increased transcription of a subunit of the CO_2_ receptor (AgGR22) is mirrored in increased neural and behavioural sensitivity to CO_2_. Behavioural activation by CO_2_ was greatest at low concentrations (600–1200 ppm). Higher concentrations in fact reduced activation of both 1 and 4 dpe female *A. gambiae*, even though the highest concentration tested was just 10% of that in human breath. The cpC-expressed receptor (AgOR28) was also significantly up-regulated. This receptor is less specific than other maxillary palp receptors, with several potential ligands identified, and their behavioural function has not yet been ascribed (Lu et al., 2007; Smallegange et al., 2012). The transcript levels of the rest of the receptor genes, *AgOrco*, *AgOr8*, *AgGr23* and *AgGr24*, were not significantly changed between 1 and 4 dpe, consistent with the functional stability of AgOR8-expressing OSNs. Although we did not test the receptor protein abundance in the neurons directly, these results show a correlation between transcript abundance and physiological activity in two different receptors, suggesting a direct relationship between transcription and the role of the ligand, decoded in behaviour under specific physiological conditions.

Activation of functionally required receptor proteins enables an efficient use of energy while amplifying a signal whose importance is relevant to a specific physiological state (Rinker et al., 2013a; Webster et al., 2015). The ORs and GRs are heteromers (Larsson et al., 2004; Sato et al., 2008), so one would expect that an equimolar presence of the receptor subunits would be necessary for optimal function (Bohbot et al., 2013). The regulation of a single subunit of the heteromeric receptors in both the OR and GR systems suggests a very simplified modulation mechanism, which changes a necessary and sufficient component to achieve down- or up-regulation of function. Lu et al. (2007) showed that *Gr22* is necessary for CO_2_ detection in *A. gambiae*, while Sengul and Tu (2008) and McMeniman et al. (2014) demonstrated that the knockdown of the orthologous gene (*Gr2*) in *A. aegypti* was sufficient to abolish CO_2_ detection. These observations are consistent with our finding. Contrary to our results, Bohbot et al. (2013) reported up-regulation of all olfactory receptors expressed in the maxillary palps of *A. aegypti* throughout maturation (1, 6 and 10 dpe) and linked this to cellular and behavioural responses. Although we used two different quantification and expression normalisation protocols, this difference would nevertheless point to an interesting biological difference between Culicine and Anopheline mosquitoes. In *Anopheles*, changes associated with host seeking appear to involve regulation of only a subset of receptors, implying that a small subset of the odour space may drive host seeking. It would be interesting to compare this among closely related species with divergent host-seeking strategies (specialists and opportunists).

Modulatory mechanisms may shape the contextual meaning of a single olfactory signal. CO_2_ has previously been reported to be associated with the host-seeking behaviour of mosquitoes (Bohbot et al., 2013; Grant and O'Connell, 2007), consistent with our observations. CO_2_ is a ubiquitous compound whose fluctuation in regular pulses above ambient is a reliable indicator of vertebrate blood hosts for mosquitoes. However, inconsistencies in the effectiveness of CO_2_-based traps to catch mosquitoes suggest that anthropophilic vectors do not depend solely on this chemical cue to locate humans (Costantini et al., 1996; Takken an Knols, 1999). Thus, an understanding of the stability of the CO_2_ plume structure and the functional role of CO_2_ will be important issues to resolve in the future. The observation that 4 day old mosquitoes were more easily stimulated to fly with ambient CO_2_ levels supports the earlier observation that host-seeking input has a non-neuronal maturation component other than the receptor function (McMeniman et al., 2014). *Aedes aegypti*, however, do not get activated by CO_2_ unless mature enough to blood feed at 6 and 10 dpe, respectively (Bohbot et al., 2013; Grant et al., 1995). Interestingly, we also observed dose-dependent behavioural response to CO_2_ in 1 dpe mosquitoes, suggesting that this compound is not restricted to blood host location. The functional and behavioural dynamic range of CO_2_ observed is consistent with concentrations that would be expected in the medium to long range following dilution of the 40,000 ppm CO_2_ exhaled by humans, for example.

The observed functional changes in receptor transcripts and neurons suggest an association of sensory signals with physiological needs. The cpB neuron expresses *AgOr8*/*AgOrco*, which showed stable transcription and unchanged sensitivity to its key ligand (*R*)-1-octen-3-ol between 1 and 4 dpe, unlike in *A. aegypti*, where it is up-regulated and the receptor sensitivity increased (Bohbot et al., 2013). As *AgOr8* is exclusively expressed in adults, this implies that transcriptional changes during pupation deliver a fully functional receptor at eclosion, and that (*R*)-1-octen-3-ol may be used at both nectar-feeding and host­-seeking stages, or that its importance at host seeking is dependent on co-detection with another compound. (*R*)-1-Octen-3-ol is also a common compound emitted by fungi (Inamdar et al., 2013). Therefore, its role in the context of sugar source seeking would be interesting to investigate.

The significant up-regulation of *AgOr28* transcripts suggests that the key ligand(s) of this receptor is important in the host-seeking behaviour of *A. gambiae.* Heterologously expressed *AgOr28* is more broadly tuned compared with *AgOr8*, responding to 2,4,5-trimethylthiazole, acetophenone, 2-acetylthiophene and fenchone, all of which are associated with mammalian odour (Carey et al., 2010; Lu et al., 2007; Xia et al., 2008). Addition of either acetophenone or 2-acetylthiophene to a basic human odour blend decreased landing of *A. gambiae* (Smallegange et al., 2012), suggesting that this receptor may be involved in mosquito host selection or discrimination.

We show that peripheral modulation may explain behavioural changes towards host seeking and demonstrate a correlation between receptor gene expression, neuronal sensitivity and behaviour. Receptor sensitivity reliably modulates the olfactory signal and contextual relevance of components of an odour plume, and might also sharpen host selection. As the maxillary palp system is a secondary olfactory organ, an investigation of the functional structure of an odour plume, involving antenna-expressed receptors, would be interesting. Such studies would also reveal suitable candidates for molecular manipulation of mosquito behaviour towards sustainable control of the diseases they transmit.

## MATERIALS AND METHODS

### Mosquitoes

*Anopheles gambiae sensu stricto* (Suakoko strain, now renamed *Anopheles colluzzi*; Coatzee et al., 2015), were reared according to standard protocols (http://www.mr4.org). Larvae were reared in plastic trays (30×15×5 cm), half-filled with distilled water, and fed every other day on Tetramin Baby fish food (Tetra GmbH, Germany). Rearing medium was refreshed with distilled water every other day. Pupae were collected into adult rearing cages (30×30×30 cm; Bugdorm, MegaView Science, Taiwan) and allowed 24 h to eclose. Adult mosquitoes were fed on 10% sucrose solution *ad libitum*. When needed for colony maintenance, adults were fed on human blood by offering a human arm for 30 min. Non-blood-fed female mosquitoes, either 1 dpe (12–24 h) or 4 dpe, were used for experiments.

### RNA extraction and qPCR

The transcript abundance of receptor genes was compared between paired 1 and 4 dpe female *A. gambiae* in six biological replicates. For each replicate, a single cohort of mosquito pupae was divided into two cages (ca. 60 pupae each) and allowed to eclose: the mosquitoes in one cage were killed the day after emergence, and those in the other cage at 4 days post-emergence to constitute a single replicate of paired treatments. Maxillary palps and proboscis of female mosquitoes from each treatment group were dissected into 300 µl Trizol (Invitrogen Corporation, Life Technologies, Carlsbad, CA, USA) and stored at −80°C until RNA extraction. All dissections occurred between 14:00 h and 16:00 h to limit potential circadian changes in gene expression. The olfactory tissues of the two treatment groups were processed side by side until the reverse transcription (RT) step to minimise variation arising from day-to-day differences, as the biological replicates and the RT step had been shown in a nested pilot study to be the greatest sources of variation. Total RNA was extracted in 500 µl Trizol reagent according to the manufacturer's protocol. The RNA pellet was washed in 70% ethanol and then in 90% ethanol, dried briefly and re-suspended in 30 µl RNAse-free water (Bio-Rad Laboratories, Inc., Hercules, CA, USA) on ice. RNA was quantified using absorbance measure (Nanodrop 2000c, Thermo Scientific, Wilmington, DE, USA) prior to DNAse treatment. Treatment with TURBO DNAse (Ambion, Life Technologies) was immediately carried out according to the manufacturer's protocol and the reaction stopped using TURBO DNAse inactivator (Ambion, Life Technologies). The supernatant was immediately used for the RT step using the iSCRIPT reaction mix (Bio-Rad Laboratories, Inc.) in three technical replicates. A 1:1 mix of oligo-dT and random hexamer primers was used, in final volumes of 20 µl each, containing 8 µl of the RNA sample. The cDNA sample was diluted three times with PCR grade water to obtain the template for qPCR assays.

### Primer design

All primers were designed using Primer 3 software (www.justbio.com) from available *A. gambiae* genome sequence information (www.vectorbase.org). All primers were designed to have a melting temperature (*T*_m_) of 60°C and a product size of 120–180 bp. Primer pairs were generally designed in adjacent exons or were intron straddling so as to exclude genomic DNA from the qPCR. Three sets of primers were designed for each target, usually in the first two exons to maximise product independent of RT efficiency. The best primer combinations were selected by analysing the specificity and compatibility of each primer set *in silico* using BLASTn and Oligoanalyser (Integrated DNA Technologies; http://eu.idtdna.com/analyzer/Applications/OligoAnalyzer). The best two combinations were tested by qPCR and a selection made by comparing the consistency of amplification in three technical replicates.

### Reference genes

*RpS7* and *RpL18* genes are the most commonly used reference genes for the quantification of transcripts in mosquito olfactory tissue (Iatrou and Biessmann, 2008; Pelletier and Leal, 2011; Sengul and Tu, 2008; Lavazec et al., 2007). However, as we found no report of the systematic testing of these genes in treatments and tissues similar to those used in this study, six other genes, commonly used in insect qPCR studies, were obtained and tested to produce the most stable combinations for this study (Bustin et al., 2009; Omondi et al., 2015) (Table 2). The expression was normalised to a reference factor comprising the geometric means of the best combination reference genes in Genex version 5 (MultiD Systems, Göteborg, Sweden).

### Quantitative real-time PCR

Quantitative PCR was done using the SYBR Green fluorescent dye for product detection. The reaction was carried out in a 20 µl reaction mix containing 10 µl iQ Supermix (Bio-Rad Laboratories, Inc.), 200 µmol l^−1^ of each primer mix, 1.5 µl cDNA sample and PCR grade water. Amplification was done on a BIORAD CFX 96 (Bio-Rad Laboratories, Inc.), using the following programme: a single 10 min cycle at 94°C, followed by 40 cycles of 12 s each at 95, 59 and 72°C. Data acquisition was done for each cycle just following each elongation step. A high resolution melting analysis (65 to 94°C in 0.5°C steps) was done to test the fidelity of the PCR. For each plate and primer set, a no-template and no-RT control was included. The transcript levels of each of the chemoreceptor genes previously shown to be expressed in the maxillary palp of *A. gambiae* (*AgGr22*, *AgGr23*, *AgGr24*, *AgOrco*, *AgOr8* and *AgOr28*; Lu et al., 2007) and of potential reference genes were assayed for each treatment.

### Single sensillum recordings

Single unit electrophysiology was performed with sharpened tungsten electrodes from the capitate peg sensilla of the maxillary palps of female *A. gambiae*, as previously described (Bohbot et al., 2010). A single set of recordings from the cpA and cpB neurons across a dose spectrum of CO_2_ and (*R*)-1-octen-3-ol, respectively, was taken from each preparation, with 10 replicates. A mounted 1 or 4 dpe female mosquito was placed in front of a continuous humidified stream of synthetic air (80% nitrogen, 20% oxygen; Strandmöllen AB, Ljungby, Sweden), which passed over the maxillary palp via a glass tube (7 mm i.d.) at 1.5 l min^−1^. Delivery of CO_2_ was regulated by two-way Teflon solenoid valves (Teddington, Skogås, Sweden) controlled via the digital output of an IDAC-4 (Syntech, Germany). The valves were connected to gas cylinders containing metered amounts of CO_2_ (150, 300, 600, 1200, 2400, 4800 ppm) and oxygen (20%), balanced by nitrogen (Strandmöllen AB). (*R*)-1-Octen-3-ol (a gift from James Logan, Rothamsted Research, UK; CAS: 3391-86-4), dissolved in GC-grade hexane (99.9% purity, Sigma-Aldrich), was used to describe the dose–response relationship of cpB. Pasteur pipettes (VWR International) containing a piece of filter paper (5×10 mm) (Whatman, GE Healthcare, UK) were loaded with 10 µl each of a (*R*)-1-octen-3-ol solution in a series of increasing concentrations (0.001–1000 ng µl^−1^). All pipettes were prepared in a fume hood and left for 30 min for the solvent to evaporate prior to use. In all experiments, insects were presented with a stimulus for 0.5 s, and pipettes were replaced between replicates.

### Behavioural assay

A glass non-choice bioassay tube, 80×9.5 cm i.d. (Majeed et al., 2014), with a laminar flow (20 cm s^−1^ wind speed) was used to assay the response of mosquitoes at each age to 380 (ambient), 600, 1200, 2400 and 4800 ppm CO_2_. CO_2_ stimulation was turned on or off manually by directing the inlet from the controller either into the bioassay tube or into the exhaust tube, to avoid pre-exposure of test animals to unintended doses of CO_2_. The CO_2_ pulses of 0.5 s on/2 s off, embedded within the background of ambient CO_2_, were generated by the stimulus controller (IDAC-4, Syntech, Kirchzarten, Germany) through two-way Teflon solenoid valves (Teddington, Lanna, Sweden) to simulate human host breath (Dekker et al., 2005). Air intake into the tube was charcoal filtered, and humidified (69–85% RH), with a pulse originating from pure CO_2_ (Strandmöllen AB) to produce the desired mix. Between each test, CO_2_ levels were monitored at the downwind end of the bioassay using a CO_2_ analyser (LI-820, LI-COR Biosciences, Lincoln, NE, USA). Wind speed and stability of flow were tested using an anemometer (ThermoAir3, Schiltknecht Messtechnik AG, Switzerland). Mosquitoes to be tested were starved (*ad libitum* access to water only) for 12 h prior the test. Females were then transferred into release cages and kept in the bioassay room for 6 h prior to use, under the same conditions as during rearing and with *ad libitum* access to water through a moist cotton ball. Release cages consisted of a Perspex tube of the same diameter as the bioassay tube, sealed at one end with 1.0 mm gauge netting and with a rotating mesh covering the door at the other end. The release cages with test insects were set into the bioassay under red light (∼280 lx) and left for ca.10 min to allow the mosquitoes to acclimatise, after which the butterfly door of the release cage was carefully opened. Testing was done between 20:00 h and 22:00 h, representing the first quarter of the scotophase. For each insect, the time taken to activation was recorded. Non-responders by 3 min were included in the analysis as censored individuals and contributed to the determination of activation levels per group. In total, 63–135 insects were tested per treatment (Table 1).

### Data analysis

Gene expression levels were determined using the ΔΔCq method (Livak and Schmittgen, 2001) on Genex Version 5 (Multi D Systems, Sweden). Gene expression levels per sample were normalised to a reference factor comprising the geometric means of the three most stable reference genes, and expressed relative to the mean of the control group (1 dpe) females. Transcription levels were compared between genes per group (1 and 4 dpe) using a two-tailed paired Student’s *t*-test implemented in Genex v5 after checking data for normality and homogeneity of residuals using Kolmogorov's test. Statistical significance values were adjusted for multiple comparisons.

Repeated measures 2-way ANOVA, followed by a Bonferroni *post hoc* test, was performed to compare the physiological activity between 1 and 4 dpe female mosquitoes with each ligand dosage using Statistica Version 8 (Statsoft, 2007). The interaction between independent variables (age and concentration) was assessed. A binomial function was used to test the proportion of mosquitoes taking flight and to calculate the confidence interval of the result for each treatment at *P*≤0.05 in R (R Development Core Team, 2011). The probability of activation function (Kaplan–Meier survivorship function) was compared among respondents using the Cox–Mantel test to test the null hypothesis that activation probability functions do not differ between pairs of age and CO_2_ activation regimes using Statistica. A censure variable was used, enabling the non-responders to contribute to the observation.
